# Supplementary figures and images for: Genome-wide analysis of retinal transcriptome reveals common genetic network underlying perception of contrast and optical defocus detection
Source: BMC Med Genomics. 2021 Jun 9;14:153. doi: 10.1186/s12920-021-01005-x (PMC8190860; doi:10.1186/s12920-021-01005-x)

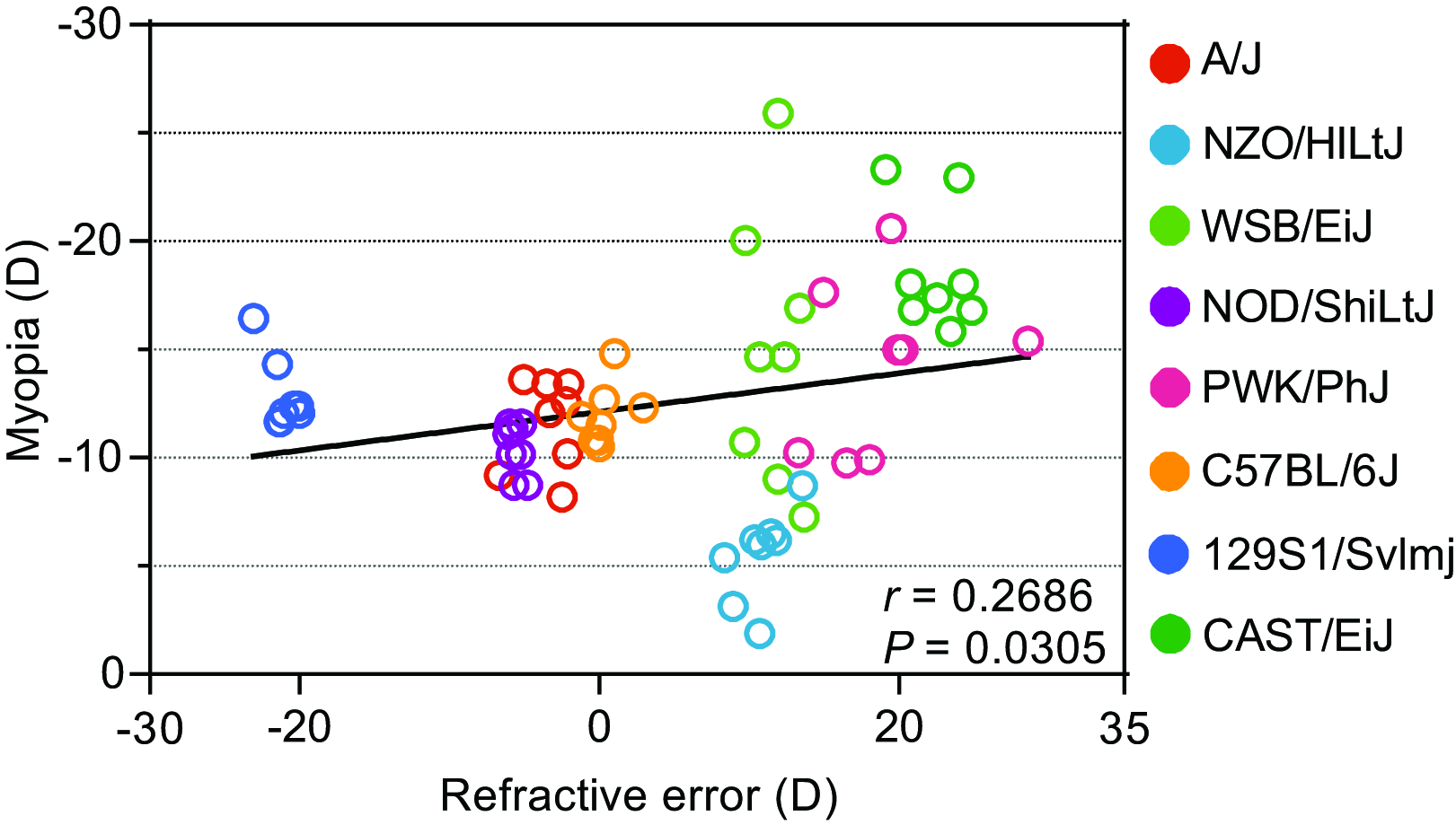

Supplement: Supplementary file 2 — Additional file 2: Figure S1. Baseline refractive error correlates weakly with susceptibility to myopia. Linear regression showing weak correlation between baseline refractive error and susceptibility to myopia. r, Pearson’s correlation coefficient; P, Pearson’s correlation significance. (TIF 729 KB) [file 12920_2021_1005_MOESM2_ESM.tif]

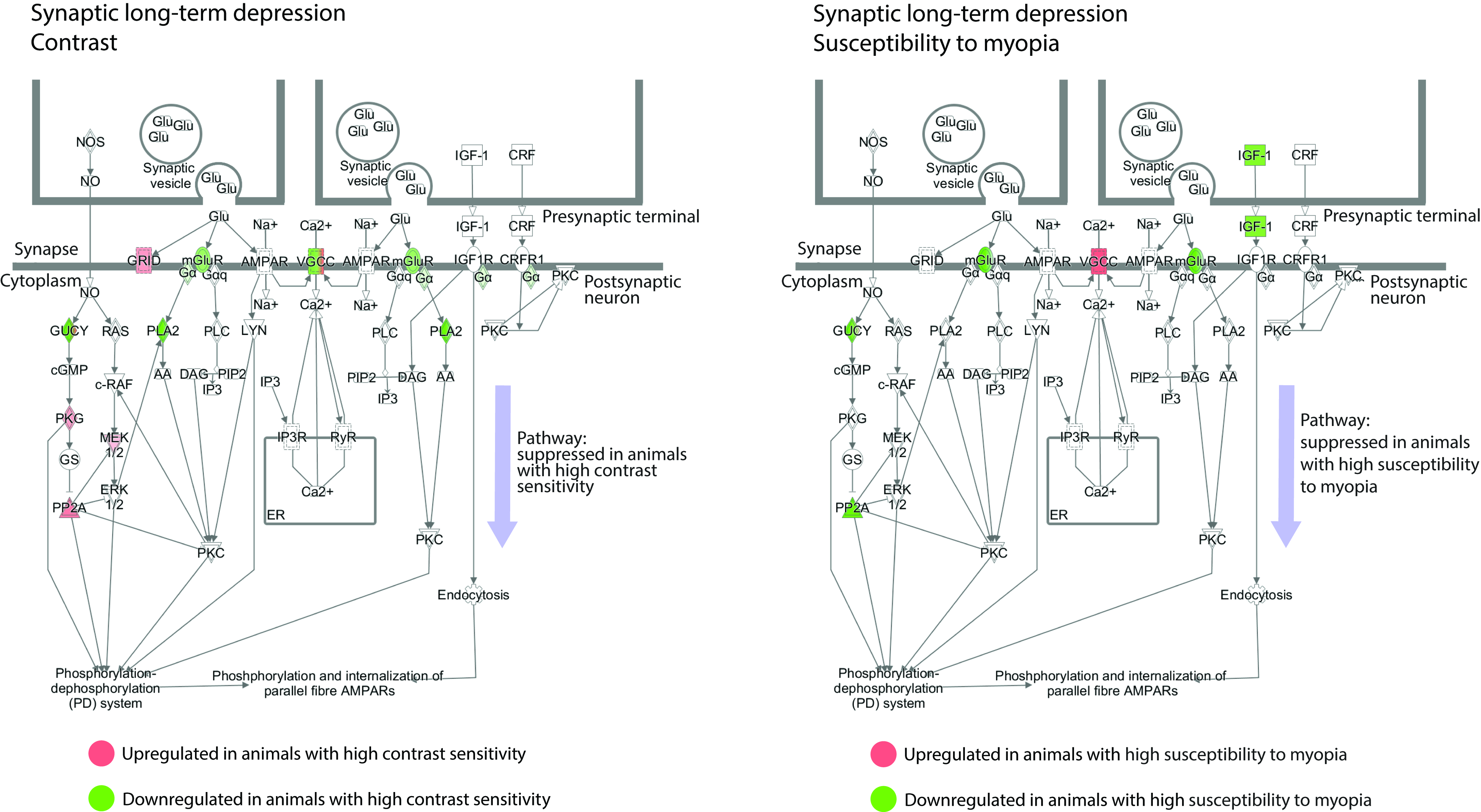

Supplement: Supplementary file 3 — Additional file 3: Figure S2. Suppression of the pathway for synaptic long-term depression leads to increased contrast sensitivity and increased susceptibility to form-deprivation myopia in mice. The diagram shows genes associated with the modulation of synaptic long-term depression in the retina and linked to both contrast perception and optical defocus detection in mice. (TIF 3156 KB) [file 12920_2021_1005_MOESM3_ESM.tif]
